# Association between early postnatal antibiotic exposure and bronchopulmonary dysplasia in very preterm infants: a meta-analysis

**DOI:** 10.3389/fped.2026.1833395

**Published:** 2026-07-16

**Authors:** Cheng Min, Ying Sui, Qu Chen

**Affiliations:** Neonatal Intensive Care Unit, Huzhou Maternity & Child Healthcare Hospital, Huzhou, Zhejiang, China

**Keywords:** antibiotics, bronchopulmonary dysplasia, meta-analysis, mortality, necrotizing enterocolitis, sepsis, very preterm infants

## Abstract

**Objective:**

Very preterm infants are at high risk of infection due to their immature immune systems, and empirical antibiotic therapy is commonly initiated shortly after birth. However, excessive or unnecessary antibiotic exposure may adversely affect organ development and long-term outcomes. This study aimed to systematically evaluate the association between early postnatal antibiotic exposure and bronchopulmonary dysplasia (BPD) as well as other adverse outcomes in very preterm infants, to provide evidence for optimizing antibiotic stewardship in neonatal care.

**Methods:**

PubMed, Embase, Wiley Online Library, and the Cochrane Library database were systematically searched from inception to March 2026. Observational cohort studies comparing early postnatal antibiotic exposure with no exposure or short-term exposure were included. The primary outcome was bronchopulmonary dysplasia (BPD), and secondary outcomes included necrotizing enterocolitis (NEC), mortality, and late-onset sepsis (LOS). Meta-analysis was performed using fixed-effects or random-effects models to calculate pooled odds ratios (ORs) with 95% confidence intervals (CIs). Sensitivity analysis and Egger's test were conducted to assess the robustness of the results and potential publication bias. For outcomes with significant publication bias, the trim-and-fill method was applied.

**Results:**

A total of 11 studies was included. Pooled analysis showed that early postnatal antibiotic exposure was associated with an increased risk of BPD (OR = 1.44, 95% CI: 1.12–1.85). In addition, the risks of NEC (OR = 1.18, 95% CI: 1.09–1.28) and mortality (OR = 1.19, 95% CI: 1.05–1.35) were significantly increased, while no statistically significant association was observed for late-onset sepsis (OR = 0.98, 95% CI: 0.71–1.34). Sensitivity analyses indicated that the results were stable. Publication bias was detected for BPD and mortality; after trim-and-fill correction, the BPD result remained robust, whereas the mortality result warrants cautious interpretation.

**Conclusion:**

Early postnatal antibiotic exposure is associated with an increased risk of BPD and several adverse outcomes in very preterm infants. Careful assessment of infection risk and optimization of antibiotic initiation and discontinuation strategies may help improve long-term outcomes in this population.

## Introduction

With the continuous advancement of perinatal medicine and neonatal intensive care technologies, the survival rates of very preterm infants (gestational age < 32 weeks) and very low birth weight infants (birth weight < 1,500 g) have significantly improved (1, 2). However, this increased survival is accompanied by a rise in long-term complications (3, 4). Among these, bronchopulmonary dysplasia (BPD) remains a major disease affecting the long-term prognosis of very preterm infants (5, 6). BPD is not only closely associated with chronic respiratory dysfunction, recurrent hospitalizations, and neurodevelopmental delays but also significantly increases the medical burden and social costs (5). Therefore, investigating its modifiable risk factors holds significant clinical importance.

Sepsis is a common complication in very preterm infants (7). Due to their immature immune function, fragile skin and mucosal barriers, and frequent invasive procedures, empirical antibiotic therapy is often administered soon after birth to prevent or control early-onset infections (6, 8). Balancing the risk of infection with the rational use of antimicrobials has become a critical clinical issue requiring urgent resolution in the field of neonatal intensive care.

In recent years, a growing body of basic and clinical research suggests that early postnatal antibiotic exposure may affect long-term organ development by disrupting the intestinal microecological balance, altering the process of bacterial colonization, and modulating inflammatory response pathways (9). The gut–lung axis theory posits that an imbalance in the gut microbiota can influence pulmonary inflammatory status and structural development through immune regulation mechanisms, potentially increasing the risk of BPD (10, 11). Furthermore, early antibiotic use may also increase the probability of necrotizing enterocolitis (NEC), late-onset sepsis (LOS), and death (12–14). These findings suggest that antibiotic exposure not only affects infectious outcomes but may also have profound impacts on multi-system development.

However, current research findings on the association between early postnatal antibiotic exposure and adverse outcomes in very preterm infants are inconsistent. Some studies report that prolonged antibiotic use is significantly associated with an increased risk of BPD and NEC, while others suggest that this association weakens or becomes non-significant after adjusting for confounding factors such as disease severity (13–17). Differences in exposure definitions, study populations, outcome criteria, and statistical methods across studies contribute to heterogeneity in the conclusions. To date, there is a lack of systematic integration of existing evidence through comprehensive analyses specifically focused on the very preterm infant population. Therefore, this study employs systematic review and meta-analysis methods to systematically integrate relevant evidence from domestic and international studies. It aims to explore the association between early postnatal antibiotic exposure and bronchopulmonary dysplasia as well as other adverse outcomes (including NEC, late-onset sepsis, and death) in very preterm infants. The goal is to provide evidence-based support for optimizing neonatal antimicrobial stewardship strategies and to offer a reference for further mechanistic research and improvements in clinical practice.

## Methods

### Study design and reporting guidelines

This study is a systematic review and meta-analysis aimed at systematically evaluating the association between early postnatal antibiotic exposure and adverse clinical outcomes in very preterm infants. The design, implementation, and reporting of this study strictly adhered to the Preferred Reporting Items for Systematic Reviews and Meta-Analyses (PRISMA) statement (18). A study protocol was predefined before implementation, including the research question, inclusion and exclusion criteria, data extraction process, and statistical analysis plan, to minimize selection bias and information bias, thereby enhancing the transparency and reproducibility of the research. This study focuses on the population of very preterm infants (typically defined as gestational age < 32 weeks), analyzing the correlation between early empirical antibiotic use and primary and secondary clinical outcomes.

### Literature search strategy

A systematic search was conducted in PubMed, Embase, Wiley Online Library, and the Cochrane Library database from inception to March 2026. The search strategy combined Medical Subject Headings (MeSH)/Emtree terms with free-text keywords, constructed around terms such as “early antibiotic exposure”, “empirical antibiotics”, “very preterm”, “very low birth weight”, “bronchopulmonary dysplasia”, “necrotizing enterocolitis”, “mortality”, “length of stay”. The search was adapted for each database according to its specific syntax. To minimize omissions, manual backward citation tracking of the reference lists of included studies was also performed. The literature search was limited to studies published in English.

### Inclusion and exclusion criteria

Included studies were required to meet all of the following criteria: (1) the study population consisted of very preterm infants (gestational age < 32 weeks) or very low birth weight (VLBW) infants (birth weight < 1,500 g); (2) clear documentation of early postnatal antibiotic exposure, typically defined as initiation of empirical antibiotic therapy within the first 72 h of life in the absence of culture-positive evidence of infection; (3) inclusion of a control group without exposure or with short-term exposure; (4) study design was an observational cohort study (prospective or retrospective); (5) reported at least one predefined outcome measure; (6) provided extractable or calculable effect estimates, including Odds Ratios (OR), Relative Risks (RR), or Hazard Ratios (HR), along with their 95% Confidence Intervals (95% CI).

Exclusion criteria were: (1) case reports, reviews, systematic reviews, or conference abstracts; (2) studies without a control group; (3) studies with incomplete data or lacking calculable effect estimates; (4) duplicate publications or studies with overlapping samples (prioritizing the study with the largest sample size or most complete data).

### Study selection and data extraction

Two reviewers independently performed study selection and data extraction. Initial screening was conducted by reviewing titles and abstracts, followed by full-text assessment of potentially eligible studies. Disagreements during the screening process were resolved through discussion and consensus, or by consultation with a third reviewer if necessary. Extracted data included: first author, publication year, study region, sample size, gestational age or birth weight range, definition of early antibiotic exposure, outcome measures, adjusted variables, and multivariable-adjusted effect estimates (OR/RR/HR) with 95% confidence intervals. If both unadjusted and adjusted results were reported, priority was given to extracting multivariable-adjusted data.

### Quality assessment

The methodological quality of the included observational cohort studies was assessed using the Newcastle–Ottawa Scale (NOS). This scale evaluates studies across three domains: Selection of study groups, Comparability of groups, and Ascertainment of Outcome, with a maximum total score of 9. Quality assessment was performed independently by two reviewers, and disagreements were resolved through discussion. Studies with an NOS score ≥7 were generally considered high-quality.

### Definition of outcome measures

The primary outcome of this study was bronchopulmonary dysplasia (BPD). BPD was defined as the need for continuous oxygen therapy or respiratory support at 36 weeks postmenstrual age. Secondary outcomes included: (1) necrotizing enterocolitis (NEC), typically diagnosed as Bell's stage ≥ II; (2) late-onset sepsis (LOS), typically defined as blood culture-positive sepsis occurring after 72 h or 7 days of age. If definitions for outcomes varied across studies, the criteria reported in the original studies were adopted, and potential clinical heterogeneity arising from these differences was considered in the analysis.

### Statistical analysis

The Odds Ratio (OR) with its 95% confidence interval was used as the primary pooled effect measure. Heterogeneity among studies was assessed using the Cochran *Q* test and the *I*^2^ statistic. An *I*^2^ value <50% indicated low to moderate heterogeneity, and a fixed-effects model was used; *I*^2^ ≥ 50% indicated substantial heterogeneity, and a random-effects model was used for pooling. To test the robustness of the results, a leave-one-out sensitivity analysis was performed. Funnel plot visual inspection combined with Egger's test was used to assess publication bias. When the funnel plot or Egger's test suggested significant publication bias, the trim-and-fill method was used to assess and adjust for its potential impact. All analyses were performed using the meta package in Stata 18MP software, and a two-sided *P*-value <0.05 was considered statistically significant.

### Quality of evidence assessment

The Grading of Recommendations Assessment, Development and Evaluation (GRADE) framework (19) was systematically used to evaluate the quality of evidence for each primary outcome. The evidence level started as “low” and was downgraded based on risk of bias, inconsistency, indirectness, imprecision, and publication bias; it could be upgraded if there were large effects, a dose-response relationship, or if residual confounding would underestimate the effect. The quality of evidence assessment was conducted independently by two researchers, with disagreements resolved through discussion or by consulting a third researcher when necessary.

## Results

### Study selection results

**Figure 1:**
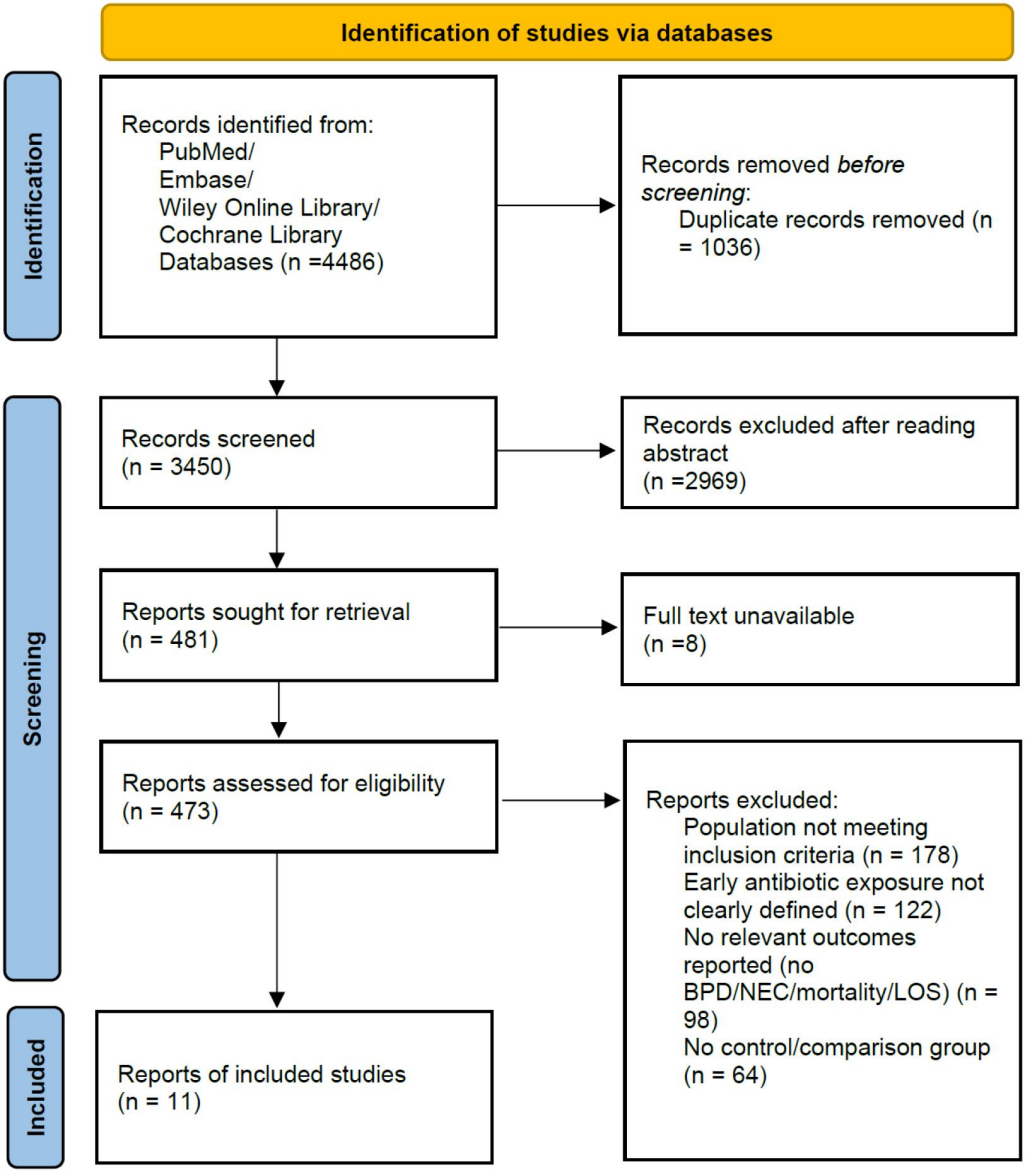
Schematic diagram of the literature screening process.

The initial database search yielded 4,486 relevant records. After removing 1,036 duplicate records, 3,450 records remained for title and abstract screening. Following title and abstract screening, 2,969 records that did not meet the inclusion criteria were excluded, resulting in 481 full-text articles being sought for retrieval. Of these, 8 were excluded because the full text could not be obtained, leaving 473 full-text articles assessed for eligibility. During the full-text assessment, 460 articles were excluded for not meeting the inclusion criteria. Ultimately, 11 studies (5, 6, 8, 12–17, 20, 21) were included in the meta-analysis. The study selection process followed the Preferred Reporting Items for Systematic Reviews and Meta-Analyses (PRISMA) flow diagram, and the specific selection process is shown in Figure 1.

### Basic characteristics of the included studies

A total of 11 observational cohort studies (5, 6, 8, 12–17, 20, 21) were included in this analysis, published between 2007 and 2023. The study regions covered multiple countries and regions across Europe, North America, and Asia. The basic information of the included studies is presented in Table 1. The sample sizes of individual studies ranged from 132 to 6,510 cases. The study population primarily consisted of very preterm infants (gestational age < 32 weeks) and very low birth weight infants (birth weight < 1,500 g). Definitions of early postnatal antibiotic exposure varied somewhat across studies, including initiation of therapy on days 0–1 after birth, antibiotic use during the first week, and stratification by duration of use (e.g., ≥5 days, >4 days, or 5–7 days vs. ≤4 days). Some studies analyzed daily antibiotic use or total days of antibiotic therapy during hospitalization as continuous variables. All studies received Newcastle–Ottawa Scale (NOS) scores ranging from 7 to 9, indicating generally high quality.

**Table 1:** Basic characteristics of the included literature.

Study (Authors, Year)	Country/Region	Period	Sample Size	Population (Gestational Age/Birth Weight)	Antibiotic Exposure (Early Week 1)	Outcome Measures	NOS
Cantey et al. 2018 (5)	USA	2010–2014	374	GA < 32 weaks/BW < 1,500 g	Any vs. None (Week 1)	NEC, Mortality, LOS	9
Chen et al. 2022 (6)	Taiwan, China	2016–2020	132	BW < 1,500 g	Per Additional Day	BPD	7
Esmaeilizand et al. 2018 (8)	Canada	2010–2014	671	BW < 1,500 g	≥5 days duration	NEC	8
Fillistorf et al. 2025 (12)	Switzerland	2007–2022	1,398	GA < 32 weaks	Daily Duration (DoA)	BPD, NEC, Mortality, LOS	8
Greenberg et al. 2019 (13)	USA	2008–2014	2,526	BW < 1,000 g	≥5 days duration	NEC, Mortality, LOS	8
Letouzey et al. 2022 (14)	France	2,011	648	GA < 32 weaks	Day 0–1 Initiation	BPD, NEC, Mortality, LOS	8
Reid et al. 2019 (15)	USA	2009–2015	328	GA < 32 weaks	≥5 days duration	Mortality	7
Shi et al. 2024 (16)	China	2019–2021	6,510	GA < 32 weaks/BW < 1,500 g	5–7 days vs. 0/1–4 days	BPD, Mortality	9
Strobel et al. 2025 (17)	USA	2015–2019	891	GA < 28 weaks	≥5 days duration	NEC	8
Vatne et al. 2023 (20)	Norway	2009–2018	5,296	GA < 32 weaks	>4 days vs. ≤4 days	BPD, NEC, Mortality, LOS	8
Zhu et al. 2023 (21)	China	2015–2018	27,532	GA < 32 weaks	≥5 days duration	NEC, Mortality	8

NOS, Newcastle–Ottawa Scale.

### Early postnatal antibiotic exposure and bronchopulmonary dysplasia

**Figure 2:**
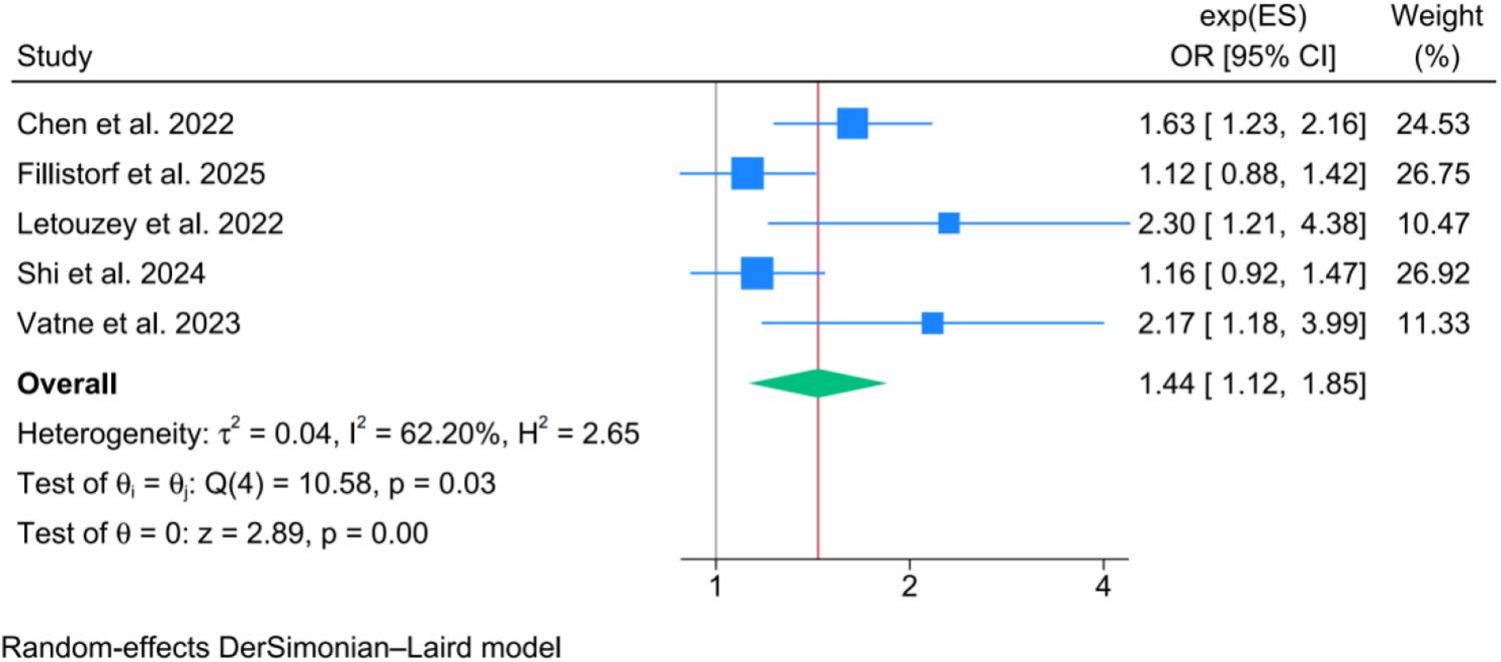
Forest plot of the meta-analysis on early postnatal antibiotic exposure and the risk of BPD.

A total of five studies (6, 12, 14, 16, 20) reported on BPD outcomes. The heterogeneity test indicated moderate heterogeneity among the studies (*I*^2^ = 62.20%, *P* = 0.03), prompting the use of a random-effects model for pooled analysis. The results demonstrated that early postnatal antibiotic exposure was associated with an increased risk of BPD, with a pooled effect size of OR = 1.44 (95% CI: 1.12–1.85), and the difference was statistically significant (*Z* = 2.89, *P* < 0.01), as shown in Figure 2.

### Early postnatal antibiotic exposure and necrotizing enterocolitis

**Figure 3:**
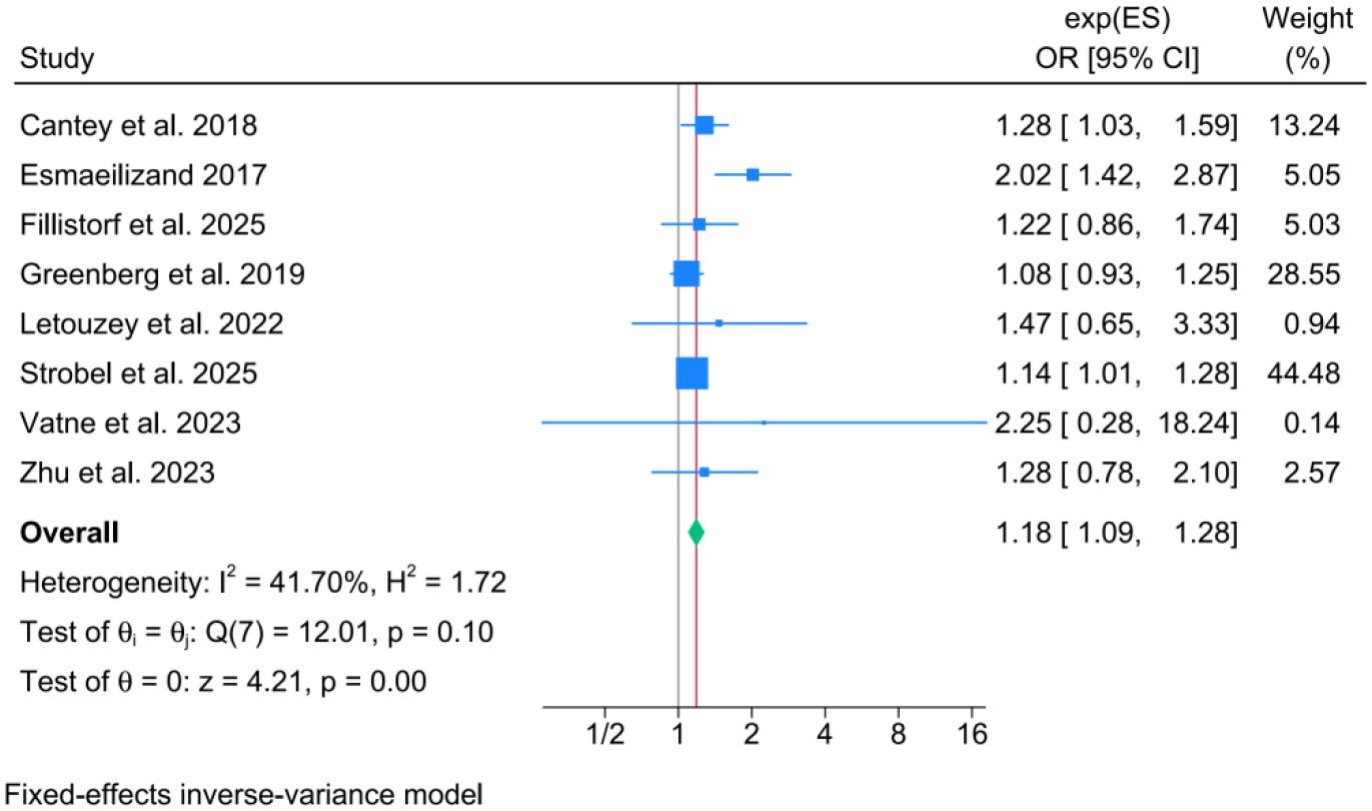
Forest plot of the meta-analysis on the association between early postnatal antibiotic exposure and the risk of NEC.

A total of eight studies (5, 8, 12–14, 17, 20, 21) reported on necrotizing enterocolitis (NEC). The heterogeneity test revealed low heterogeneity among the studies (*I*^2^ = 41.70%, *P* = 0.10), so a fixed-effects model was used for pooled analysis. The results showed that early postnatal antibiotic exposure was associated with an increased risk of necrotizing enterocolitis, with a pooled effect size of OR = 1.18 (95% CI: 1.09–1.28), and the difference was statistically significant (*Z* = 4.21, *P* < 0.01), as shown in Figure 3.

### Early postnatal antibiotic exposure and mortality

**Figure 4:**
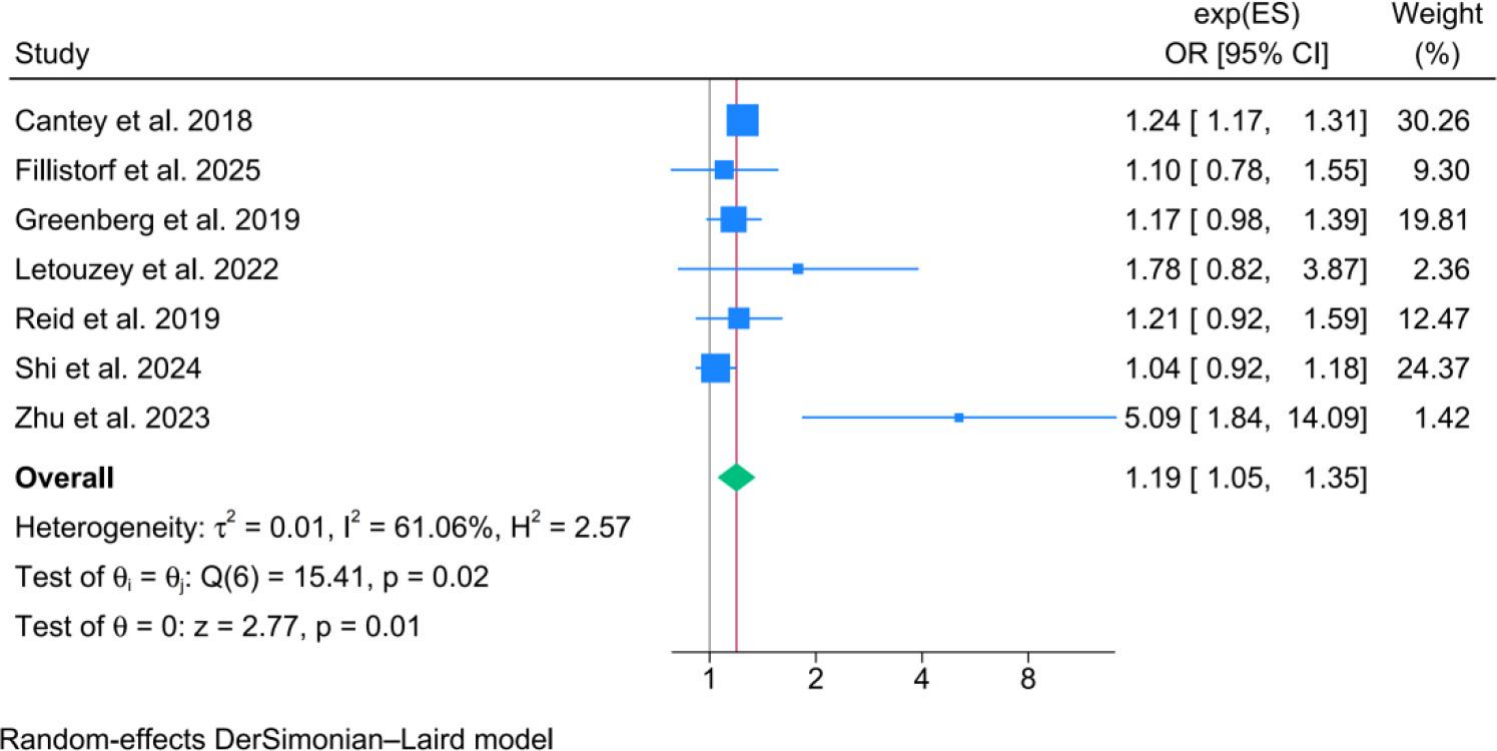
Forest plot of the meta-analysis on early postnatal antibiotic exposure and the risk of mortality [after exclusion of Vatne et al. (20)].

A total of eight studies (5, 12–16, 20, 21) reported mortality outcomes. When Vatne et al. was included, the pooled OR for all eight studies was 1.21 (95% CI: 1.05–1.38), with moderate-to-high heterogeneity (*I*^2^ = 62.74%, *P* = 0.01). Given that Vatne et al. reported an OR of 9.33 (95% CI: 1.10–79.5) with a wide confidence interval, which could disproportionately influence the pooled estimate, the primary analysis was based on the exclusion of this study. After exclusion, moderate heterogeneity was observed among the remaining seven studies (*I*^2^ = 61.06%, *P* = 0.02), and a random-effects model yielded a pooled OR of 1.19 (95% CI: 1.05–1.35, *Z* = 2.77, *P* < 0.01), as shown in Figure 4.

### Early postnatal antibiotic exposure and late-onset sepsis

**Figure 5:**
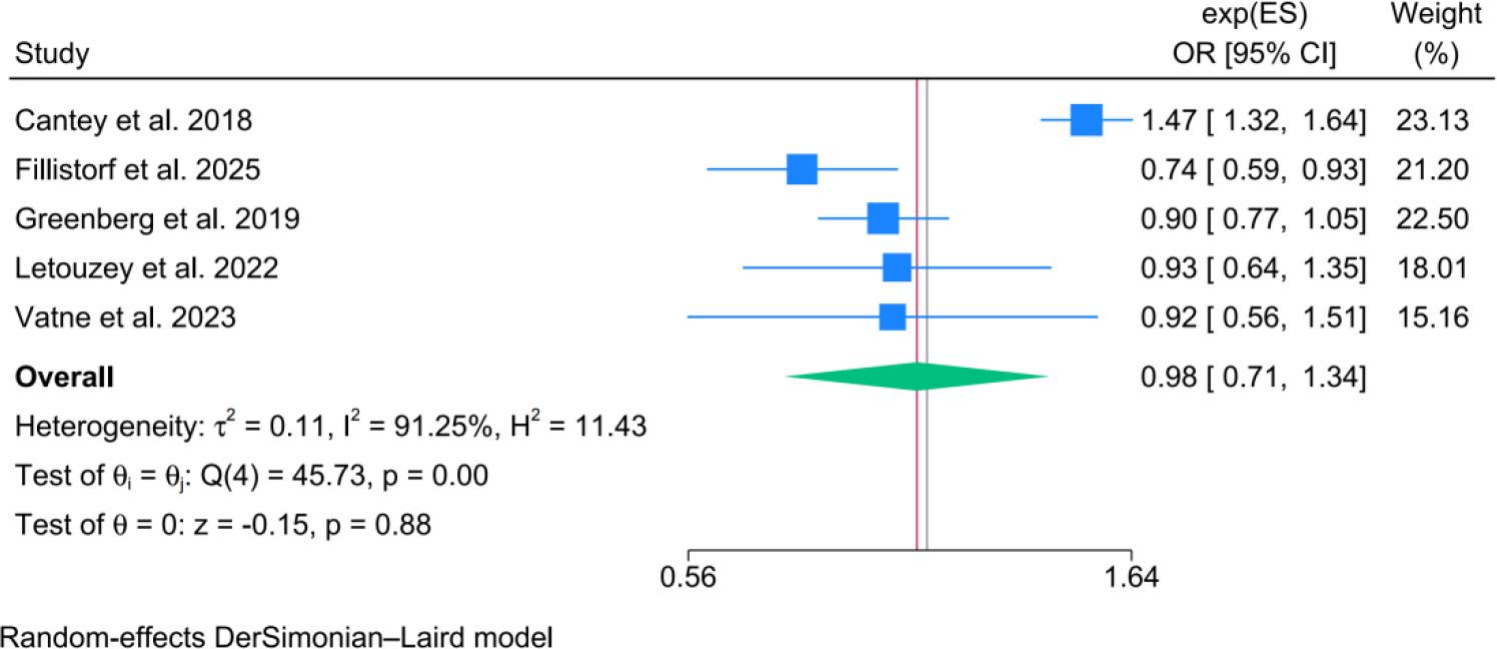
Forest plot of the meta-analysis on the association between early postnatal antibiotic exposure and risk of LOS.

A total of five studies (5, 12–14, 20) reported LOS outcomes. The heterogeneity test indicated high heterogeneity among the studies (*I*^2^ = 91.25%, *P* < 0.01), therefore a random-effects model was used for pooled analysis. The results showed that early postnatal antibiotic exposure was associated with an increased risk of late-onset sepsis, with a pooled effect size of OR = 0.98 (95% CI: 0.71–1.34), and the difference was not statistically significant (*Z* = −0.15, *P* = 0.88), as shown in Figure 5.

### Sensitivity analysis and publication bias

Sensitivity analysis, performed by sequentially excluding individual studies, revealed no significant alterations in the results, indicating that the overall findings were robust. Additionally, for the mortality outcome, Vatne et al. was excluded from the primary analysis to reduce its disproportionate influence. Publication bias was assessed using Egger's regression test. The results showed that the Egger's tests were not statistically significant for NEC and LOS (both *P* > 0.05), suggesting no significant publication bias for these outcomes. For BPD and mortality, Egger's tests indicated significant publication bias (both *P* < 0.05), and the trim-and-fill method was applied to assess the potential impact of unpublished studies. For BPD, the pooled estimate decreased from 1.441 to 1.365 after trim-and-fill, but the direction and statistical significance remained unchanged, indicating robust results. For mortality, the 95% confidence interval of the pooled estimate crossed 1 and the statistical significance changed after trim-and-fill, suggesting that this result may be influenced by publication bias and requires further validation. Due to the limited number of included studies (<10), particularly for LOS, the statistical power for publication bias assessment was limited, as shown in Table 2.

**Table 2:** Egger's test results.

Outcome indicator	Number of studies	*Z* value	*P* value	Before trim-and-fill	After trim-and-fill
Risk of BPD	5	2.706	0.007	1.441 [1.125, 1.845]	1.365 [1.070, 1.741]
Risk of NEC	8	1.793	0.073	NA	NA
Risk of Death	7	2.18	0.030	1.191 [1.052, 1.349]	1.154 [0.995, 1.338]
Risk of LOS	5	−0.705	0.481	NA	NA

### Quality of evidence assessment

Based on the GRADE framework, the quality of evidence for the primary outcomes was assessed. The results showed that the certainty of evidence f was very low for BPD and mortality, low for NEC, and very low for LOS. Overall, the quality of evidence for each outcome was primarily affected by the risk of bias due to observational study designs, between-study heterogeneity, publication bias for BPD and mortality, and the limited number of studies included for LOS. Publication bias was detected for BPD and mortality, while no significant publication bias was observed for NEC and LOS. The GRADE assessment suggests that the findings of this study have certain reference value, but high-quality prospective studies are still needed for further validation, as shown in Table 3.

**Table 3:** GRADE quality of evidence assessment for each outcome.

Outcome indicator	Study design	Risk of bias	Inconsistency	Imprecision	Publication bias	Certainty of evidence
Bronchopulmonary dysplasia (BPD) association	Observational cohort study	Serious	Serious	No serious	Serious	⊕⊝⊝⊝ Very Low
Necrotizing enterocolitis (NEC) association	Observational cohort study	Serious	No serious	No serious	No serious	⊕⊕⊝⊝ Low
Mortality association	Observational cohort study	Serious	Serious	No serious	Serious	⊕⊝⊝⊝ Very Low
Late-onset sepsis (LOS) association	Observational cohort study	Serious	Serious	Serious	No serious	⊕⊝⊝⊝ Very low

## Discussion

This systematic review and meta-analysis indicates that early postnatal antibiotic exposure is associated with multiple adverse outcomes in very preterm infants, with the association with BPD being the most robust. Pooled analysis suggested that early antibiotic exposure was associated with an increased risk of BPD (OR = 1.44, 95% CI: 1.12–1.85), which was statistically significant. Considering the high proportion of empirical antibiotic use in the very preterm infant population, coupled with the relatively limited incidence of true culture-positive infections, this finding suggests that early antibiotic exposure may not merely be a marker of disease severity but could also participate pathophysiologically in the development of BPD.

The pathogenesis of BPD is not attributable to a single factor but results from the interplay between arrested lung development and sustained inflammatory stimulation (6, 9, 22). Alveolar development in very preterm infants is still in the canalicular or saccular stage, rendering the lung structure highly immature and particularly susceptible to inflammation and oxidative stress (23, 24). Recent research has progressively shifted from the “mechanical ventilation and oxygen toxicity injury” model towards an “inflammation-driven developmental disruption” model, emphasizing the long-term impact of early systemic inflammatory load on alveolarization and pulmonary vascular development (14–16). Consequently, the effect of antibiotics on the gut microbiota has emerged as a new research focus. Early antibiotic exposure may affect alveolarization and pulmonary vascular development by disrupting intestinal microecological balance, reducing microbial diversity, delaying the colonization of dominant flora, and amplifying systemic inflammatory responses; this mechanism is consistent with the current understanding of BPD pathophysiology and the gut–lung axis (25).

Under normal conditions, the gut microbiota establishes rapidly within days after birth and participates in the maturation of the immune system. Early postnatal antibiotic exposure can significantly reduce microbial diversity, delay the establishment of dominant bacteria such as Bifidobacteria, and promote the proliferation of opportunistic pathogens, leading to a state of persistent microbial dysbiosis (15, 26). Alterations in the gut microbiota can influence systemic immune homeostasis by modulating T-cell differentiation, the release of inflammatory cytokines, and levels of metabolites (e.g., short-chain fatty acids). This regulatory pathway, known as the “gut-lung axis,” provides a biological explanation for the link between antibiotic exposure and BPD: perturbation of the gut ecosystem may amplify systemic inflammatory responses, thereby affecting the processes of alveolarization and pulmonary vascular remodeling (17). Furthermore, antibiotics themselves may also impact mitochondrial function and oxidative stress pathways. Certain antimicrobial agents can interfere with host mitochondrial protein synthesis or affect energy metabolism. Their long-term effects, particularly in the developmentally immature neonatal population, remain not fully elucidated (16, 17, 20). Therefore, early postnatal antibiotic exposure may create an unfavorable environment for lung development through the convergence of multiple mechanisms.

This study also observed an increased risk of NEC, which is considered a typical disease associated with microbiota imbalance, involving alterations in gut flora, intestinal mucosal barrier damage, and excessive inflammatory responses (14, 15, 20). A clinical co-occurrence between NEC and BPD has also been noted, potentially forming an “inflammatory cascade” through systemic inflammatory amplification effects and multi-organ interactions (15–17). Therefore, the increased risk of NEC and the elevated risk of BPD may share common pathological pathways to some extent, rather than being independent of each other. Pooled results for mortality suggested an increased risk, but these findings require cautious interpretation. The administration of antibiotics in very preterm infants is often based on infection risk assessment, and their use may itself reflect the severity of the underlying disease, such as lower gestational age, higher respiratory support requirements, or increased risk of perinatal infection (5). Therefore, the association between antibiotic exposure and mortality may be partly attributable to differences in underlying disease burden, and the mortality results in this study should be interpreted as “associations” rather than definitive causal effects. Furthermore, significant publication bias was detected for mortality. The trim-and-fill analysis showed that the 95% confidence interval of the pooled estimate crossed 1 after correction, indicating that this result may be influenced by publication bias and warrants cautious interpretation. On the other hand, prolonged or unnecessary antibiotic use may also adversely affect infant outcomes by disrupting intestinal microecological balance, increasing colonization of drug-resistant bacteria, and promoting secondary infections (12, 13). Previous studies have shown that reduced gut microbiota diversity may increase susceptibility to neonatal infections (14, 20). Furthermore, LOS itself is a common severe complication during hospitalization in very preterm infants, and its occurrence is closely related to various factors, including invasive procedures, central venous catheterization, mechanical ventilation, and prolonged hospital stay (12–14). Early antibiotic exposure may alter microbial colonization patterns, facilitating the colonization of opportunistic pathogens in the gut or on the skin and mucosa, thereby increasing the likelihood of late-onset infections. However, due to differences across studies in the definition of LOS, infection diagnostic criteria, and stratification of antibiotic exposure, considerable heterogeneity was observed in the analysis of this outcome. Furthermore, given that the LOS outcome in this study did not reach statistical significance, this mechanistic explanation requires further validation.

Notably, moderate to high statistical heterogeneity was observed for the outcomes of mortality (*I*^2^ = 61.06%) and late-onset sepsis (*I*^2^ = 91.25%), indicating differences across studies in population characteristics, exposure definitions, stratification by antibiotic duration, and respiratory support strategies. For instance, some studies used “initiation of antibiotics” as the exposure criterion, while others used “duration ≥5 days” for grouping, which are not entirely equivalent in terms of exposure intensity. Additionally, some studies did not strictly exclude cases with early-onset infection or culture-positive infants, which may have further influenced effect estimates. The aforementioned factors may all influence heterogeneity. However, due to substantial variations across included studies in the definition of antibiotic exposure, stratification of treatment duration, population composition, and outcome criteria, it was difficult to establish consistent and comparable stratification standards. Therefore, no further subgroup analyses or meta-regression were conducted in this study. Additionally, in the meta-analysis, the weight distribution of different studies was not balanced: sample size was positively correlated with the proportion of overall effect, and small-sample studies contributed less to the pooled estimate. Consequently, the overall conclusions of this study were primarily influenced by studies with larger sample sizes and more stable estimates. Finally, this study is an observational analysis of risk factors, and the OR is primarily used to measure the strength of the relative association between exposure and outcome, rather than absolute risk changes in interventional trials. Thus, the interpretation of results should focus on the direction and strength of the association, rather than making direct causal inferences. Previous cohort studies have also suggested a potential association between early antibiotic exposure and adverse outcomes such as BPD or death, although the findings across studies are not entirely consistent (25). Furthermore, significant publication bias was detected for the BPD outcome, and the certainty of evidence for NEC and mortality was low, while that for BPD and LOS was very low in the GRADE assessment. Nevertheless, the sensitivity analysis and trim-and-fill results indicated that the direction and statistical significance of the BPD association remained unchanged after correction, suggesting that the core finding is not substantially driven by publication bias alone. Therefore, while the quality of evidence is limited by both heterogeneity and potential publication bias, the overall conclusion regarding the BPD risk remains supported by the available data. Despite these limitations, this study has certain strengths. First, the included studies were generally of high quality with a broad range of sample sizes, enhancing the stability of the results. Second, by focusing on BPD as a core outcome for pooled analysis, the research question was addressed with greater clarity. Third, the comprehensive assessment of multiple outcomes facilitates a systematic understanding of the potential impacts of antibiotic exposure.

In conclusion, the findings of this study suggest that in very preterm infants, early postnatal antibiotic exposure is associated with an increased risk of developing BPD, along with significantly increased risks of NEC and mortality. Given the imbalance in weight distribution across studies and the inconsistency in outcome definitions, further high-quality prospective, multicenter studies are still needed for validation, with efforts to distinguish the effects of early antibiotic exposure *per se* from confounding factors attributable to the severity of underlying diseases. Meanwhile, considering the widespread use of antibiotics in clinical practice, future efforts should focus on optimizing the criteria for initiating and discontinuing empirical antibiotic therapy while ensuring infection safety, and strengthening antimicrobial stewardship strategies.

## Data Availability

The original contributions presented in the study are included in the article/Supplementary Material, further inquiries can be directed to the corresponding author.
